# Non-native three-dimensional block copolymer morphologies

**DOI:** 10.1038/ncomms13988

**Published:** 2016-12-22

**Authors:** Atikur Rahman, Pawel W. Majewski, Gregory Doerk, Charles T. Black, Kevin G. Yager

**Affiliations:** 1 Center for Functional Nanomaterials, Brookhaven National Laboratory, Upton, New York 11973, USA

## Abstract

Self-assembly is a powerful paradigm, wherein molecules spontaneously form ordered phases exhibiting well-defined nanoscale periodicity and shapes. However, the inherent energy-minimization aspect of self-assembly yields a very limited set of morphologies, such as lamellae or hexagonally packed cylinders. Here, we show how soft self-assembling materials—block copolymer thin films—can be manipulated to form a diverse library of previously unreported morphologies. In this iterative assembly process, each polymer layer acts as both a structural component of the final morphology and a template for directing the order of subsequent layers. Specifically, block copolymer films are immobilized on surfaces, and template successive layers through subtle surface topography. This strategy generates an enormous variety of three-dimensional morphologies that are absent in the native block copolymer phase diagram.

## Abstract (web summary)

Molecules can self-assemble to form ordered phases with defined shape and periodicity, but there are a limited set of morphologies reported. Here, the authors have manipulated block copolymer thin films via an iterative assembly process to form a diverse library of morphologies.

Self-assembly has emerged as a prominent paradigm for the controlled formation of nanoscale-ordered phases^1^,^2^,^3^. Block copolymer (BCP) thin films provide a straightforward means of rapidly and spontaneously generating uniform nanomaterials over macroscopic areas, where polymer architecture reliably encodes the nanoscale structural motif^4^,^5^. However, the inherent energy-minimization aspect of self-assembly emphasizes shapes that minimize surface area and maximize symmetry. Self-assembly thus yields a very limited set of morphologies, such as lamellae or hexagonally packed cylinders^6^,^7^. Here, we overcome this challenge, and demonstrate how soft self-assembling materials can be manipulated to form a diverse library of previously unreported morphologies. Non-equilibrium morphologies are accessed using a pathway-dependent assembly strategy. Our paradigm uses iterative assembly of BCP films, where each layer acts as both a structural component in the final morphology and a template that guides subsequent self-assembling layers. This is different from other layered-assembly approaches: one extreme is the simple stacking of independently ordered layers (layer-by-layer)^8^,^9^,^10^,^11^,^12^,^13^,^14^, which squanders the adaptive nature of self-assembly; on the other extreme is the direct replication of the first-layer pattern by subsequent layers (epitaxy)^15^,^16^,^17^, which cannot access non-native morphologies. Here, we present an intermediate ‘responsive layering' approach that leverages the adaptive nature of soft materials, with each layer responding in a controlled fashion to those underneath. This strategy generates an enormous variety of three-dimensional (3D)morphologies that are not native to the bulk equilibrium BCP phase diagram.

## Results

### Responsive layering method

**Figure 1:**
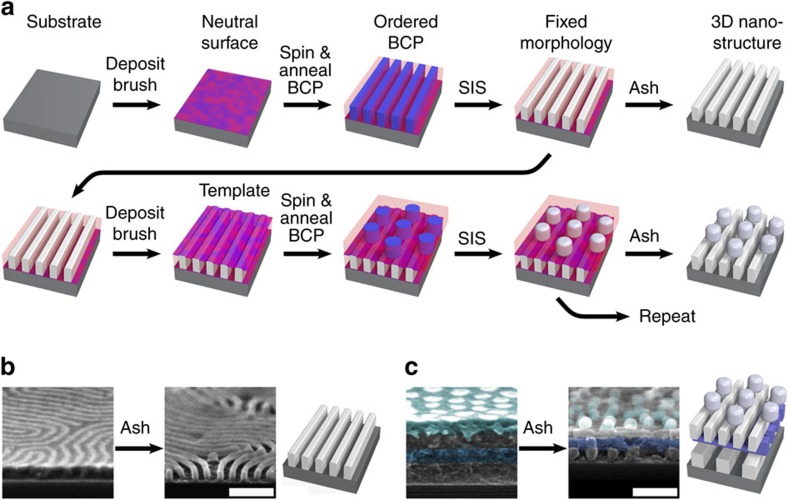
Morphology assembly scheme. (**a**) The responsive assembly strategy begins by depositing a neutral brush on the substrate of interest. A block copolymer (BCP) film is then spin-coated on the substrate and annealed to yield a well-defined morphology. Selective infiltration synthesis (SIS) is used to load one of the BCP domains with alumina; this process ‘fixes' the thin film, making it robust and insoluble, and generates height variation between the domains. The fixed BCP film can be used as the substrate in a subsequent round of ordering. The height variation (which remains after depositing the neutral brush) templates the subsequent BCP layer, causing it to align and register in a well-defined way. Single-layer, bi-layer or multilayer inorganic replicas can be formed by ashing the film (exposure to O_2_ plasma), as shown in the right-most column. (**b**) Cross-sectional scanning electron microscopy (SEM) of an exemplar single-layer infiltrated nanostructure before and after ashing (lamellar-forming BCP, L36). (**c**) Cross-sectional SEM of an exemplar four-layer nanostructure. False-colour applied to highlight layers (from bottom to top): L104, L74, L36, C132. Scale bars are 100 nm.

Our approach relies on sequential ordering and immobilization of two-dimensional (2D) BCP thin films in order to construct 3D morphologies in a layered fashion (Fig. 1). We spin-cast and anneal polystyrene-*block*-poly(methyl methacrylate) diblock copolymers (PS-*b*-PMMA) to yield ordered nanoscale phases^5^,^18^,^19^,^20^, and selectively infiltrate the PMMA block with alumina using a vapour-phase precursor (trimethylaluminium)^21^,^22^. This selective infiltration synthesis (SIS) serves two roles: the infiltration ‘fixes' the BCP film, rendering it insoluble and allowing additional polymer film applications atop; and preferential loading of the PMMA block with alumina swells this phase, generating subtle surface topography coincident with the morphology (1–4 nm; Supplementary Fig. 1). The fixed polymer-alumina composite film thus acts as a substrate for overlying assembly steps, leveraging the BCP's tendency to self-align and register to underlying surface features. We spin-cast a neutralizing brush^19^,^23^,^24^,^25^, which prevents direct chemo-epitaxial replication of the underlying BCP pattern. The thin (∼6 nm) brush coats conformally and preserves the surface topography (Supplementary Figs 1–4). Thus, each BCP layer orders in response to the underlying topography, which is itself defined by self-assembly. This responsive layering can be repeated multiple times, building up a 3D morphology in a layered fashion. The entire structure can be converted into an inorganic alumina replica by ashing (exposure to O_2_ plasma, Fig. 1b,c). This methodology can rapidly generate intricate 3D nanostructures, including structures possessing cavities or voids in underlying layers (e.g., Fig. 1c).

### New morphologies

**Figure 2:**
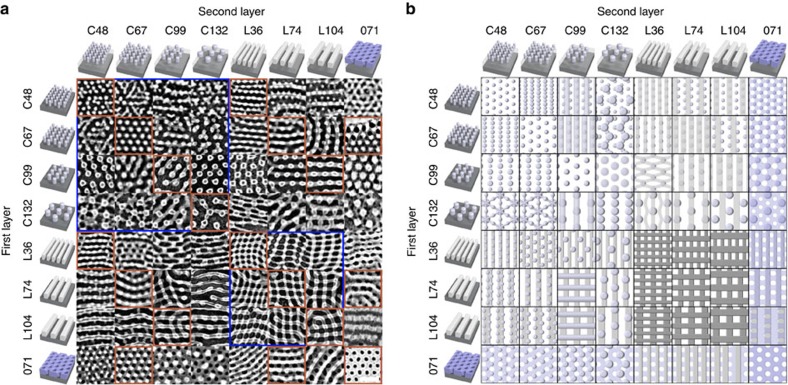
Diversity of morphologies. (**a**) Scanning electron microscopy (SEM) images of two-layer nanostructures formed by the iterative assembly of block copolymer films. Scale bar is 100 nm, and applies to all images. The ordering of the second layer is templated by the morphology of the first layer, giving rise to combined morphologies not native to the bulk equilibrium BCP phase diagram. The blue boxes highlight areas where the same class of morphology is used in both layers; the red boxes highlight conditions where the lattice spacings of the two layers are roughly equal. (**b**) Schematics of the idealized three-dimensional assemblies formed, highlighting the enormous diversity of morphologies that can be accessed.

This approach makes possible the creation of an enormous diversity of combined morphologies by marrying together BCP layers with different phases (cylinders, C; lamellae, L; and inverse-cylinders, O) and intrinsic repeat-periods (L_0_, which is dictated by the copolymer molecular weight). Figure 2 shows scanning electron microscopy (SEM) images and schematics for all bilayer combinations assembling eight representative BCP materials (samples are designated by MXX, where M is the morphology and XX is the molecular weight). In addition to structures that resemble known BCP morphologies (e.g., hexagonally packed dots), a host of new morphologies appear. The most straightforward two-layer example is the successive applications of the same cylinder-forming material, where the second layer of vertical cylinders self-registers epitaxially to the first (e.g., C67–C67). The combined structure thus exhibits the same symmetry as the component materials (p6mm symmetry, with respect to the projected 2D order). The successive application of the same morphology can also yield structures distinct from the components. For lamellae, the second-layer material preferentially anti-aligns with respect to the first, giving rise to square (e.g., L74–L74, p4mm symmetry) or rectangular (e.g., L36–L104, p2mm symmetry) patterns. Combinations of disparate morphologies can give rise to new surprising structures. For instance, ordering of lamellar L74 on top of the hexagonal C48 material yields stripes interposed through every second pair of cylinder rows, with the cylinders forming a ‘zig-zag' motif (p2mg symmetry). The library of bilayer morphologies obtained by combining cylinder, lamellar and inverse cylinders shows a significantly greater degree of complexity compared with the three simple component morphologies (Fig. 2b and Supplementary Fig. 5). The combined morphologies include examples of 7 different wallpaper symmetry groups (out of the 17 possible), and display significant structural diversity within a particular symmetry group (Supplementary Fig. 6). Importantly, the combined morphologies frequently exhibit symmetry and structural motifs not present in either of the constituent layers (Supplementary Fig. 7), emphasizing that the obtained structures are self-assembled and emergent.

**Figure 3:**
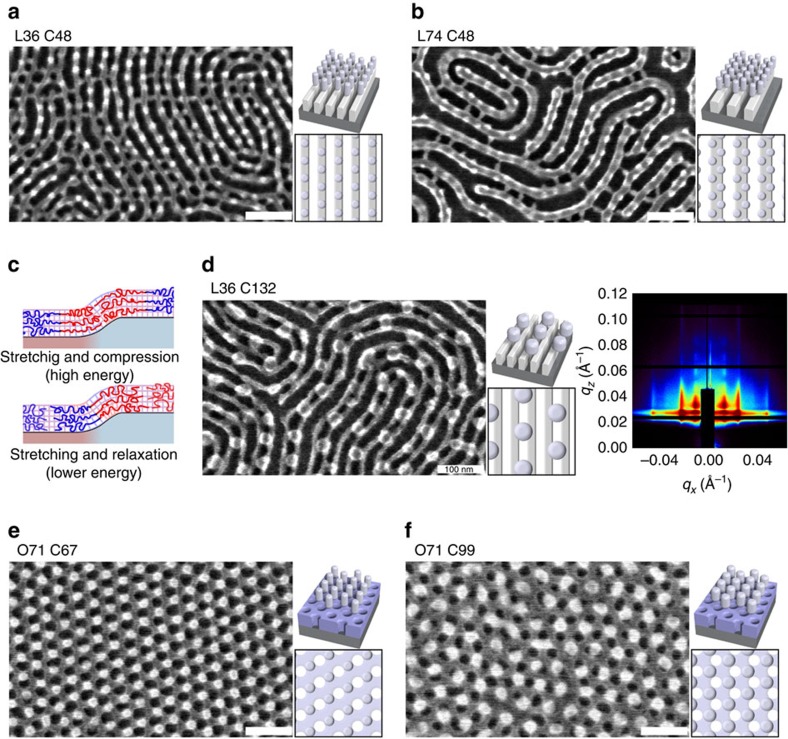
Templated ordering. (**a**) Commensurate assembly of a cylinder material atop a lamellar involves the cylinders organizing along the underlying stripes. (**b**) When an incommensurate cylinder phase orders on top of a larger repeat-period lamellar pattern, the cylinder row-spacing distorts so as to align the cylinders with the edges of the underlying stipes. (**c**) This registry phenomena can be understood in terms of chain distortion. If the central region of a BCP domain is positioned on top of a height variation, the BCP chains must stretch and compress. If instead the interblock interface is positioned over the height variation, some BCP chains stretch, while other stretched chains are unstretched (relax), leading to a lower overall energy. (**d**) This registration can be seen in C132 ordering atop L36, where the edges of cylinders align with underlying stripes. Corresponding X-ray scattering demonstrates the templated order over wide areas. (**e**) Nearly-commensurate cylinders ordering atop a honeycomb pattern results in two offset hexagonal lattices, again maximizing overlap of the interblock interface with the height variation. (**f**) Slightly incommensurate ordering exhibits similar registry, but with higher defect density. Scale bars are 100 nm.

A key feature in the observed assembly is the well-defined registry between successive layers, even when combining disparate morphologies. For instance, when C48 assembles on L36, the cylinder cores are positioned exactly on top of the underlying PMMA/alumina stripes (Fig. 3a). Assembly of C48 on top of larger repeat-period lamellae results in the second-layer cylinders aligning on the edge between the PMMA/alumina and PS regions, resulting in zig-zag patterns (Fig. 3b). It is clear that the second layer is not ordering independently from the first. For instance, when a large repeat-period cylinder phase (C132) orders on a small repeat-period lamellar pattern (L36), the second-layer cylinders register in-between the first-layer stripes, occupying every other interstice, and maintaining local hexagonal cylinder packing (Fig. 3d). This ordering is conserved over macroscopic sample dimensions, as confirmed by Fourier transforms of wide-area SEM images (Supplementary Figs 8 and 9) and grazing-incidence X-ray scattering (Fig. 3e and Supplementary Figs 10–12). The registration of layers requires distortion of the second-layer repeat-period when the two materials are not commensurate. For instance, L74 stretches its period to register within every second row of a C48 hexagonal lattice, adopts its bulk equilibrium period to align with the rows of C67 material and stretches to remain 1:1 overlapped with the larger spacing of C99 and C132 (confirmed using Fourier analysis; Supplementary Fig. 9). Overall, the registered self-assembly of two-layer nanostructures gives rise to a host of non-native morphologies (see Supplementary Figs 13–20 for examples). For instance, the organization of L36 on C99 (or C132) gives rise to undulating stripes that ‘cross-connect' between cylinders. Fourier analysis confirms the suppression of the pure L36 ordering, and the emergence of a distinct combined unit cell (Supplementary Figs 13–15).

### Mechanism of responsive layering

We understand this unique ordering behaviour by noticing that the second BCP layer tends to maximize the overlap of its interblock interfaces to the interblock interfaces in the underlying layer. Owing to the SIS-induced swelling of the PMMA domain, the interblock interface is where maximum height variation arises. Thus, the second-layer BCP orders so as to overlap its interblock region with the underlying height variation. In the L36–C48 assembly, the second-layer cylinders align with the first-layer stripes to maximize the overlap of the cylinder edges with the stripe edges. For L104–C48, the C48 domains sit just off the edges, with the stripes running in between, as this maximizes the overlap of the C48 cylinder perimeters with the underlying height variation. This registration phenomenon can be explained in terms of BCP chain stretching (Supplementary Discussion and Supplementary Figs 21–24). When a BCP film orders on a weak (<L_0_) topographic pattern, it will bend to conformally coat the substrate, generating an internal stress field. Bending involves a combination of polymer chain stretching and compression (Supplementary Fig. 22). The chains within an ordered BCP mesophase are stretched, with the segments near the interblock interface more stretched than those nearer the chain ends^26^,^27^,^28^,^29^. Bending a BCP in the centre of one of the domains involves stretching and compressing relatively unperturbed chains; both of these distortions incur an energy penalty. In contrast, bending a BCP at the interblock domain boundary involves distorting chains that are already highly stretched. Further stretching involves an additional energy penalty; however, compression of stretched chains relaxes them toward their unperturbed conformation (Supplementary Fig. 21). Thus, the overall energy penalty for distorting the BCP domain boundary is less than distorting other regions of the morphology. This results in the BCP organizing so as to maximize the overlap between substrate height modulations and the domain boundary region of the morphology, since this overall lowers the chain distortion energy penalty (Fig. 3c).

This heuristic explains the rich behaviour observed in combining a wide variety of morphologies and size scales. For example, lamellar domains orient perpendicularly to underlying lamellar morphologies (Fig. 2)^30^,^31^,^32^. This ‘crossed' alignment completely eliminates stretching/compression of the polymer chains along the long axis of the coil (i.e., normal to the lamellar interblock interface). In this perpendicular orientation, the lamellar chains can accommodate the underlying height variation by simple rearrangement of chain packing orthogonal to the coil long axis. Such a simple accommodation is not possible for cylinder morphologies, since polymer chains are arranged across all in-plane orientations. For cylinder second layers, the morphology orients and registers so as to maximize the overlap of the interblock interface with underlying height variation (to minimize chain distortion along the coil long axis). Cylinder-forming material (C67 or C99) ordering atop a slightly incommensurate honeycomb pattern (O71) shifts so as to overlap cylinder perimeters with the underlying height variation, resulting in two offset hexagonal lattices (Fig. 3e,f). Greater mismatch in period can introduce a relative rotation of the two lattices (Supplementary Fig. 20).

BCP assembly is known to be responsive to confinement^33^,^34^,^35^, guiding trenches^36^,^37^,^38^ (i.e., grapho-epitaxy^39^,^40^), topographic disruptions^41^,^42^,^43^,^44^, and substrate roughness^45^,^46^, corrugations^30^,^31^,^32^ or faceting^47^,^48^,^49^. BCPs typically respond according to commensurability, reorienting and distorting to preserve their bulk equilibrium morphology. Our work suggests a subtler variant, where the BCP not only distorts to be commensurate with the underlying pattern but also registers to weak topography in order to minimize chain perturbation. The full diversity of structures we observe (Fig. 2) can be explained using a cascade of chain distortion effects: the repeat-period may distort to become commensurate with the under-layer; the morphology adopts an orientation and registry to minimize chain distortion by placing interblock interfaces atop height variations; finally, in cases of degenerate configurations, the material with a lower bending energy penalty will sit on top of height variations. These effects are evidently strong enough to distort the BCP far from its bulk equilibrium morphology (c.f. undulating lines of C99–L36 or bridging-bulges of C132–C67). It is also possible that for some morphologies, the final development steps (infiltration and ashing) play a role in accentuating, or selecting, the observed features.

Film thickness influences the ordering phenomena observed herein. It is well-known that BCP films are strongly responsive to film thickness^1^,^4^,^5^,^50^,^51^,^52^, with the morphology or orientation being influenced by interfaces and commensurability considerations (Supplementary Fig. 25). For the results presented here, the film thickness was monolayer or sub-monolayer (with respect to the BCP repeat-distance), which drives ordering towards a vertical orientation of the morphology (cylinder long axis perpendicular to substrate; lamellar sheets perpendicular to substrate). This ordering propensity can be confirmed by noting that the first layer in the bilayer morphologies (Fig. 2) all exhibit vertical orientations. The same spin-casting conditions were used for the second layer of the bilayer structures. In general, these second-layer materials also adopt a vertical orientation. However, a number of counterexamples can be seen (e.g., C67–C99), which indicates that the responsive layering phenomenon observed here is sufficiently strong to overcome thin film confinement effects. The orientation and order (defect density) of the second-layer material can be tuned by adjusting the film thickness. Thus, film thickness is an additional parameter that can be used to control ordering of multilayer BCP nano-structures.

The presented energy model ignores many pertinent aspects of BCP response, including interplay with film thickness, and the potential formation of different morphologies to minimize chain distortion. These possibilities could be captured by more sophisticated modelling. Field-theoretic methods have emerged as a powerful tool for understanding the assembly of BCP phases^6^, and it is likely that their application to the materials described herein could yield new insights. For instance, self-consistent field theory was used to demonstrate that relatively weak surface corrugations could strongly influence BCP assembly, selecting among different possible morphologies^49^. These simulations help to confirm the heuristic presented here. That is, subtle substrate topography can control the registration of a BCP film. Simulations of BCP ordering under more extreme topographic confinement predict the appearance of 3D motifs very distinct from bulk ordering^53^,^54^,^55^,^56^, suggesting that elaborated extensions of the methods presented herein will yield an even greater diversity of nanostructures.

An important consideration in assessing self-assembling nanomaterials is their defectivity. Conventional BCP materials (single-component thin film) exhibit substantial defect densities. The topological defects in BCP phases (disclinations, dislocations, grain boundaries) are high energy^57^,^58^; at equilibrium the expected areal defect density would be very low. The much larger defect densities observed experimentally are thus metastable, arising from the ordering history, rather than equilibrium fluctuations^59^. While the local ordering (morphology) is determined thermodynamically, the defectivity is kinetic. We observe similar phenomena in our multilayer assembled morphologies. The local ordering motifs can be ascribed to equilibrium effects, where the layer relaxes into the (local) energy minimum. On the other hand, the defect density is kinetically limited (e.g., can be reduced with annealing time). In our responsive layering scheme, defects can arise in three different ways. Firstly, the BCP materials exhibit the same inherent defectivity as conventional single-layer BCP films; thus each layer will exhibit a certain density of kinetically limited defects. Additionally, the second layer will have a higher defect density, since it inherits defects from the first layer; that is, any defect in the first layer will disrupt the preferred local motif, introducing a defect in the second layer. Finally, incompatibility between the morphologies in subsequent layers may frustrate ordering, generating new defects; for instance, incommensurability will cause an overlayer to distort in order to register to underlying height variations, with the associated internal stress increasing the probability of defects. Indeed, we observe that commensurate combinations (e.g., O71–C67) exhibit long-range order, while incommensurate combinations have increased defect densities.

The acceptable level of defectivity of course depends on the target application. For some applications, long-range order is crucial, whereas for others, well-defined local morphology is sufficient. For example, tuning bulk optical and electrical properties, or generating membranes for nano-filtration or catalysis, requires optimization of the local nanoscale structure, but is insensitive to long-range order. On the other hand, applications in nano-electronics or data storage require exceptionally low defect densities over large areas. The materials presented herein should immediately be useful for a wide variety of nano-materials applications. Where lower defectivity is necessary, the presented assembly strategy could easily be combined with established directed self-assembly methods, which can rapidly generate aligned BCP phases with considerably lower defectivity^20^. An exciting avenue for future investigation is to study the interplay between directed self-assembly methods and the responsive layering described herein.

The presented mechanistic explanation provides means of predictably generating desired three-dimensional nanostructures. Combining commensurate BCP materials (1:1, 1:2, etc.) will minimize defectivity, and will give rise to nanostructures where the structures of the individual phases are preserved and well-registered to one another, thus forming a distinct overall symmetry. Combining slightly incommensurate layers will give rise to new, distorted morphologies (e.g., undulating lines), while highly incommensurate combinations may yield poorly ordered structures. Generally speaking, a size-mismatch between the nanostructures in the two layers will give rise to ‘out-of-phase' combined structures, where the second-layer structure (e.g., cylinder cores) sit *in between* the first-layer structures. Conversely, layering of similar-sized nanostructures gives rise to ‘in-phase' combinations, where the structures in the layers align on top of one another. We note that the mechanism is not dependent on system-specific properties, and should thus manifest for any BCP materials. Indeed, we confirmed similar behaviour in the assembly of polystyrene-*block*-poly(2-vinyl pyridine) BCPs (Supplementary Fig. 26).

## Discussion

**Figure 4:**
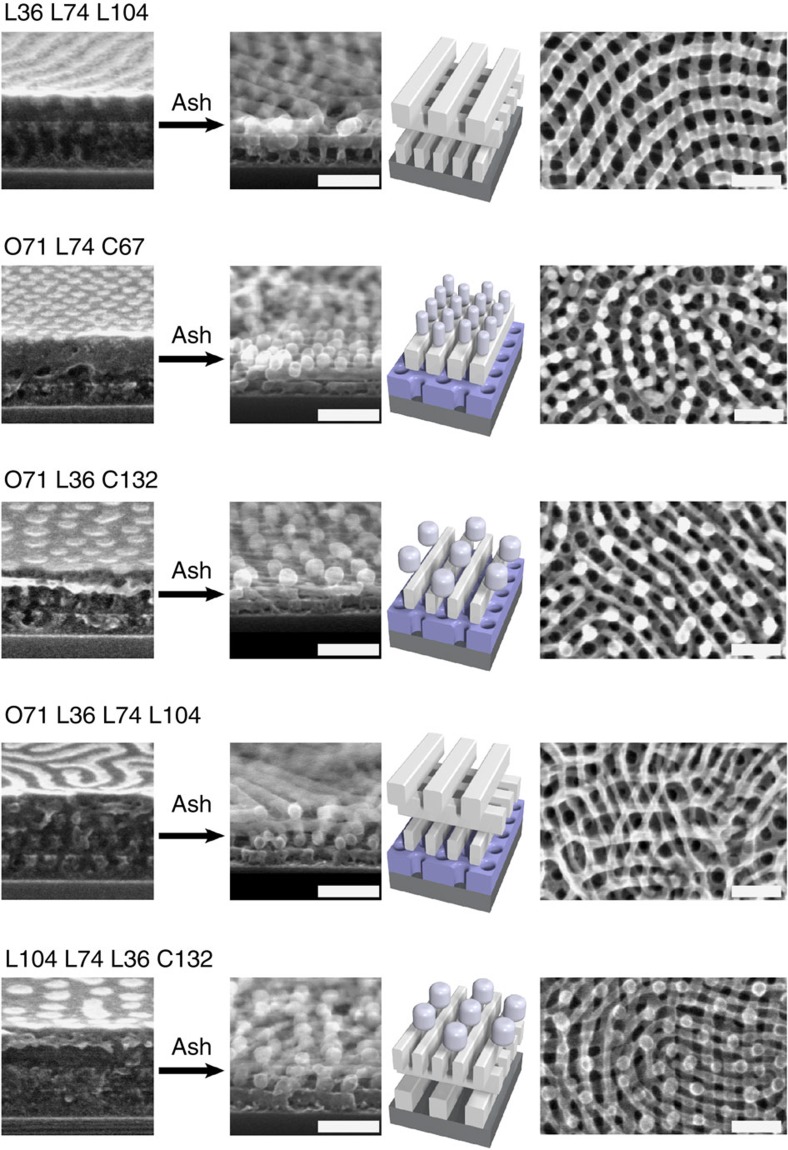
Multilayered 3D nanostructures. Examples of 3D nanostructures formed by layered block copolymer assembly. The order of layers (from bottom to top) is given for each row. The post-ash materials are clearly nanoporous, with underlying channels and voids. Scale bars are 100 nm.

The presented method allows the rapid formation of an enormous diversity of 3D morphologies. There has been substantial progress in expanding the range of self-assembled shapes through materials synthesis (e.g., triblocks^60^,^61^,^62^) and preparation (e.g., blending^61^,^63^,^64^). Directed assembly using lithographic templates can also promote the formation of distinct patterns^42^,^65^,^66^,^67^. Our iterative self-assembly method allows morphologies to be explored rapidly and combinatorially, by simple layering of existing materials. Conventional layer-by-layer or epitaxial approaches allow straightforward prediction and control of structure formation, but sacrifice many of the inherent advantages of self-assembly. Conversely, our method exploits the adaptive nature of self-assembly to generate surprising non-equilibrium morphologies. Our responsive layering paradigm can construct a wide variety of complex morphologies over wide areas, including three-dimensional nanostructures containing underlying channels or pores (Fig. 4). At each layer, the ordering occurs spontaneously, resulting in a controlled, templated structure; thus, one can select whether underlying features are exposed or covered. We note that it would be challenging to construct similar structures using conventional self-assembly, or even advanced lithographic techniques. This level of control opens the door to a wide variety designed materials, and thus broadens the range of applications that can be targeted.

## Methods

### Polymer materials

Polystyrene-*block*-poly(methyl methacrylate) (PS-*b*-PMMA) block copolymers of various molecular weights (ranging from 48 to 132 kg mol^–1^) were obtained from Polymer Source Inc.; a subset of materials were characterized by nuclear magnetic resonance spectroscopy, and gel permeation chromatography to confirm composition and purity. Throughout this work, we use a sample code MXX, where M denotes the morphology (L, lamellar; C, cylinder phase with PMMA as minority; O, inverse cylinder phase with PMMA as matrix) and XX denotes the total molecular weight (in kg mol^–1^). For example, C48 refers to a cylinder-forming PS-*b*-PMMA (PS matrix, PMMA cylinder cores) of total molecular weight 48 kg mol^–1^ (31.6 kg mol^–1^ PS, 17.5 kg mol^–1^ PMMA, polydispersity *M*_w_/*M*_n_=1.06), which exhibits a repeat-period of *L*_0_=26 nm.

### Substrates

In order to control the domain orientation relative to the substrate and air interfaces, silicon substrate surfaces were neutralized by applying a PS-*r*-PMMA-OH random copolymer (provided by the Dow Chemical Company), which acts as a brush that is chemically ‘neutral' (similar interaction energy with PS and PMMA). The brush composition was 61 mol% styrene, determined by ^13^C NMR, with total molecular weight *M*_n_=9.2 kg mol^–1^ and polydispersity *M*_w_/*M*_n_=1.35 (determined by gel permeation chromatography relative to PS standards). The neutral brush was applied by spin coating at 750 r.p.m., followed by annealing at 220 °C for 5 min to promote adhesion and finally rinsed in toluene to remove excess material. This protocol results in a deposited brush layer of ∼6 nm (based on spectroscopic ellipsometry).

### Polymer films

BCP thin films were prepared by spin casting 1% (wt/wt) toluene solution of PS-*b*-PMMA onto polished silicon (100) substrates with neutral brush coating. These coating conditions yield a polymer film thickness of 20–30 nm (depending on material), which is in the monolayer or sub-monolayer regime. A consistent spin-casting protocol was used for all the layers described in the main text. This enables unambiguous cross-comparison of results. We note, however, that the ordering of a particular multilayered morphology could be optimized by adjusting the film thicknesses of the various layers. BCP samples were annealed at 220 °C for 5 min on a hot plate to yield ordered morphologies. Cylindrical phase PS-*b*-PMMA (∼70:30 PS:PMMA weight ratio) self-assemble into locally ordered hexagonal arrangements of PMMA cylinders within a PS matrix (denoted C). Owing to the neutral substrate, the cylinder long axis is oriented perpendicular to the substrate plane. Opposite tone or inverse-cylinder PS-*b*-PMMA with 20:51 PS:PMMA weight ratio (O71 material) generates hexagonal patterns of PS cylinders in PMMA matrix. Finally, ∼50:50 weight ratio PS-*b*-PMMA generates lamellar (L) morphologies, which orient with the lamellar sheets perpendicular to the substrate plane (owing to the neutral brush) and thus appears as line/space (‘fingerprint') patterns. As a control experiment, a subset of bilayer morphologies were ordered with different annealing conditions (varying temperature and/or annealing time). In such cases, the final local ordering motifs (morphology of the multilayer nanostructure) were found to be identical, although the defect density was variable. This suggests that the structure formation is driven by equilibrium effects (energy minimization), while defectivity is a result of kinetic trapping and slow coarsening kinetics (precisely consistent with well-known single-layer BCP thin film ordering results).

### Material conversion

BCP films were immobilized by exposure to vapour phase precursors. Polymer films can alternatively be immobilized via crosslinking^8^,^9^,^16^,^17^,^68^,^69^; however, a key feature of our method is to exploit infiltration to generate surface height variation coincident with the morphology. The PMMA regions of the self-assembled BCP patterns were transformed into metal oxide nanostructures using SIS; that is, by sequential exposure to a suitable vapour-phase organometallic precursor and water vapour^21^,^22^. In our case, samples were exposed to four sequential cycles of trimethylaluminium followed by water vapour, using Cambridge Nanotech atomic layer deposition tool (2 min exposures to ∼10 Torr of both trimethylaluminium and water at 85 °C) to convert the PMMA regions into aluminium oxide. The infiltration of PMMA regions causes swelling of these domains, leading to the formation of a 1–4 nm topography coincident with the morphology (as confirmed by scanning probe microscopy).

### Multilayers

To fabricate multilayered structures, the infiltrated samples were used as a substrate for the above-described process. Crucially, the infiltrated BCP is robust and insoluble, allowing spin-coating from toluene to be used to deposit additional layers. Thus, the infiltrated BCP material has a neutral brush deposited using spin-coating and baking, followed by deposition of an additional BCP layer by spin-coating and annealing. This multilayered system can be infiltrated and used again as the substrate for further layering. The neutral brush deposited onto an infiltrated BCP layer is uniform, despite the chemical inhomogeneity of the BCP top surface. This was confirmed based on AFM height measurements (refer to Supplementary Fig. 1), where the height variation of a brush-coated material (2–4 nm) is less than the expected thickness of the brush layer (6–7 nm). This likely occurs because the brush material is able to adsorb to both PS and PMMA/AlO_*x*_ regions, and may be facilitated by a partial and ultra-thin AlO_*x*_ layer that incidentally coats even the PS regions (owing to a generic ALD process). Such an ultra-thin layer would decrease chemical inhomogeneity, while not impeding eventual removal of the PS domains via ashing.

### Ashing

After multilayer structure fabrication, the organic BCP templates were removed using oxygen plasma etching (20 W, 100 mTorr, for 3 min at room temperature). This process removes the organic material, leaving behind an inorganic replica of the ordered PMMA domains. Multilayered materials can be ashed with the same protocol; SEM imaging confirms that organic material is removed from all layers.

### Imaging

Samples were imaged using SEM in a Hitachi S-4800 instrument. Surface topography was quantified using scanning probe microscopy in intermittent-contact (‘tapping') mode, at 1 Hz scanning rate, on an Asylum MFP-3D instrument. Scanning probe microscopy tips (MikroMash HQ:NSC14/Al BS) were Si, 8 nm tip radius, with resonant frequency 160 kHz (spring constant 5.0 N m^–1^).

### GISAXS

Grazing-incidence small-angle X-ray scattering^70^ measurements were performed at the 11-ID CHX (Coherent Hard X-ray) beamline of the National Synchrotron Light Source II, at Brookhaven National Laboratory. Samples were measured under vacuum using an X-ray beam of 8.8984, keV (*λ*=0.1393, nm). Grazing-incidence small-angle X-ray scattering data presented were collected across a range of incidence angles (0.13–0.21°). Silver behenate powder was used as a standard for data conversion to *q*-space. Beam size was approximately 20 μm × 20 μm, thereby probing a film area of (20 μm) × (6.7 mm)≈0.1 mm^2^ in grazing-incidence geometry.

### Data availability

The data that support the findings of this study are available from the corresponding author upon reasonable request.

## Additional information

**How to cite this article:** Rahman, A. *et al*. Non-native three-dimensional block copolymer morphologies. *Nat. Commun.* **7,** 13988 doi: 10.1038/ncomms13988 (2016).

**Publisher's note**: Springer Nature remains neutral with regard to jurisdictional claims in published maps and institutional affiliations.

## Supplementary Material

Supplementary Information Supplementary Figures, Supplementary Discussion, and Supplementary References
