# Supplementary material for: Non-native three-dimensional block copolymer morphologies
Source: Nat Commun. 2016 Dec 22;7:13988. doi: 10.1038/ncomms13988 (PMC5196037; doi:10.1038/ncomms13988)
Supplement: Supplementary Information — Supplementary Figures, Supplementary Discussion, and Supplementary References [file ncomms13988-s1.pdf]

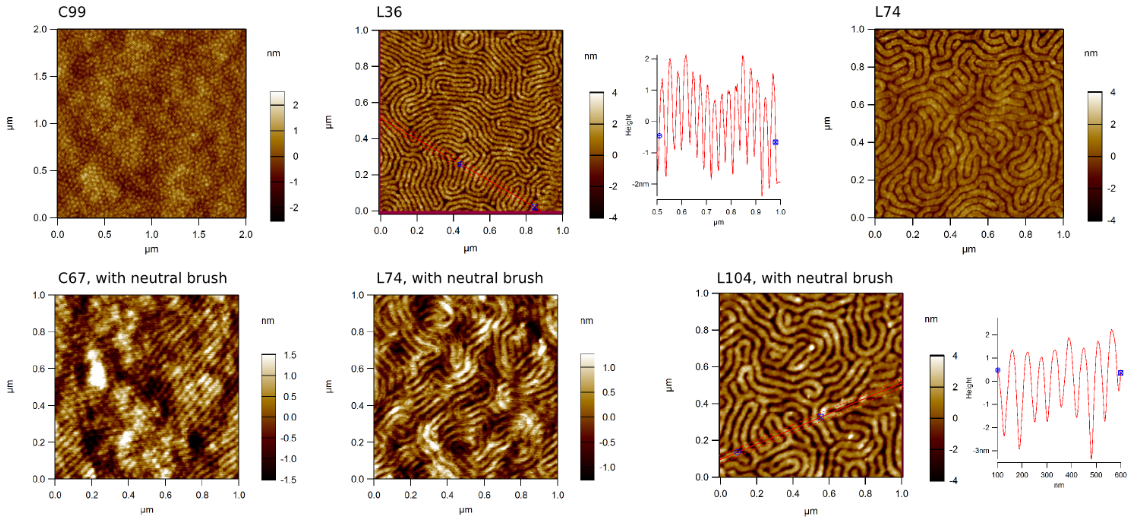

Supplementary Figure 1: Height images from scanning probe microscopy (SPM) acquired in intermittent-contact ('tapping') mode. Linecuts through the image are shown in two cases. The height variation of an infiltrated BCP film is $1-4\,\mathrm{nm}$. After deposition of the neutral brush, significant height variation remains ($2-4\,\mathrm{nm}$).

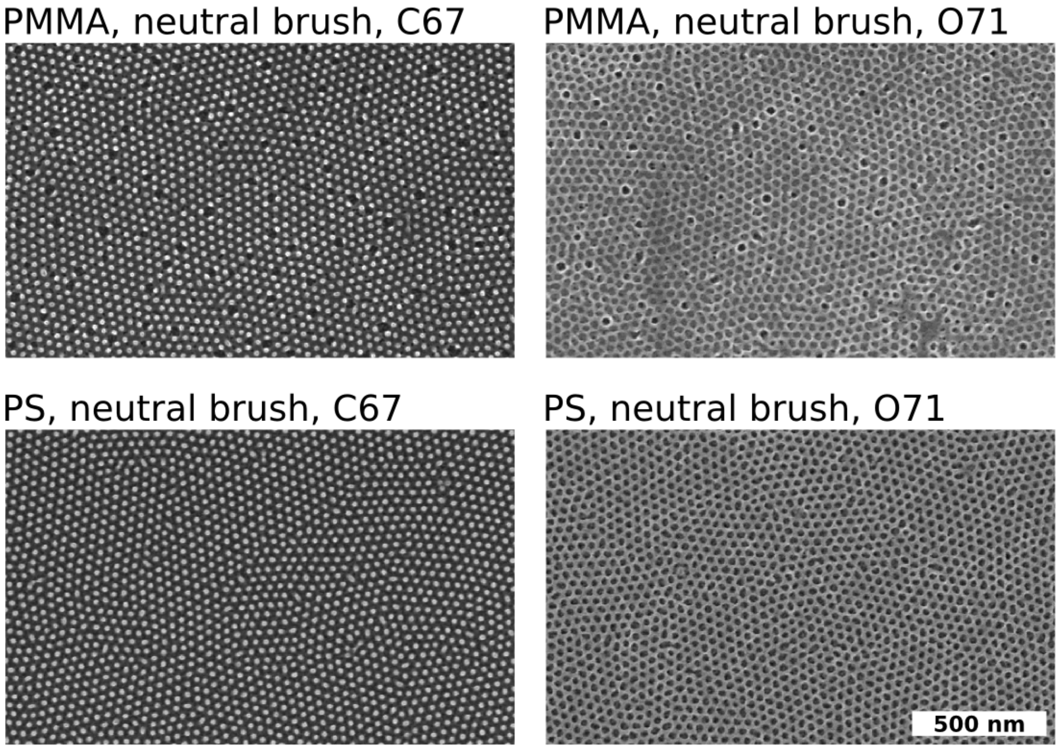

Supplementary Figure 2: Examples of morphologies ordered on neutral brushes. In the provided examples, two different BCP morphologies (cylinder phase C67, and inverse-cylinder O71) are ordered on top of substrates coated with neutral (random copolymer) brushes. Both PMMA-coated and PS-coated substrates (after SIS infiltration) can be successfully and fully neutralized by casting a neutral brush layer on top. This further implies that the brush does not allow the chemical heterogeneity of PS-*b*-PMMA underlayers to influence the registry of subsequent layers (whereas the underlying surface topography is reproduced by the brush).

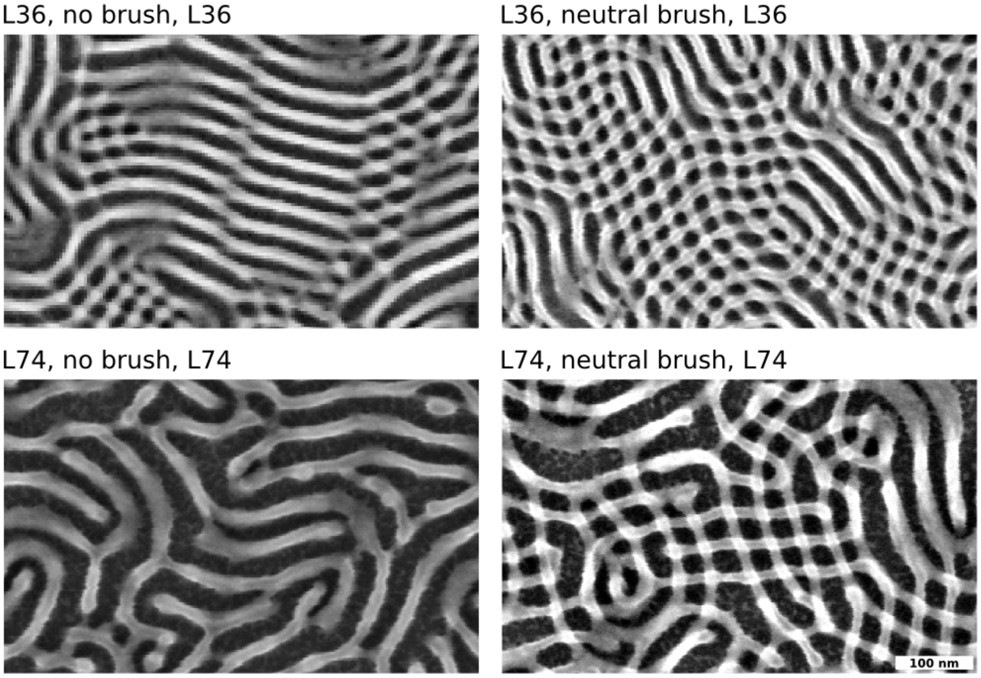

Supplementary Figure 3: Examples of two-layer morphologies ordered with or without an interlayer of chemically-neutralizing brush (random copolymer of PS and PMMA). Without a neutral brush (left), the second-layer lamellar material preferentially orders on top of the first layer material (i.e. the second-layer PMMA domains preferentially wet the underlying PMMA/AlOx regions). With a neutral brush (right), the second-layer materials instead order in response to the topography (which, for lamellae-on-lamellae leads to anti-alignment of the second layer).

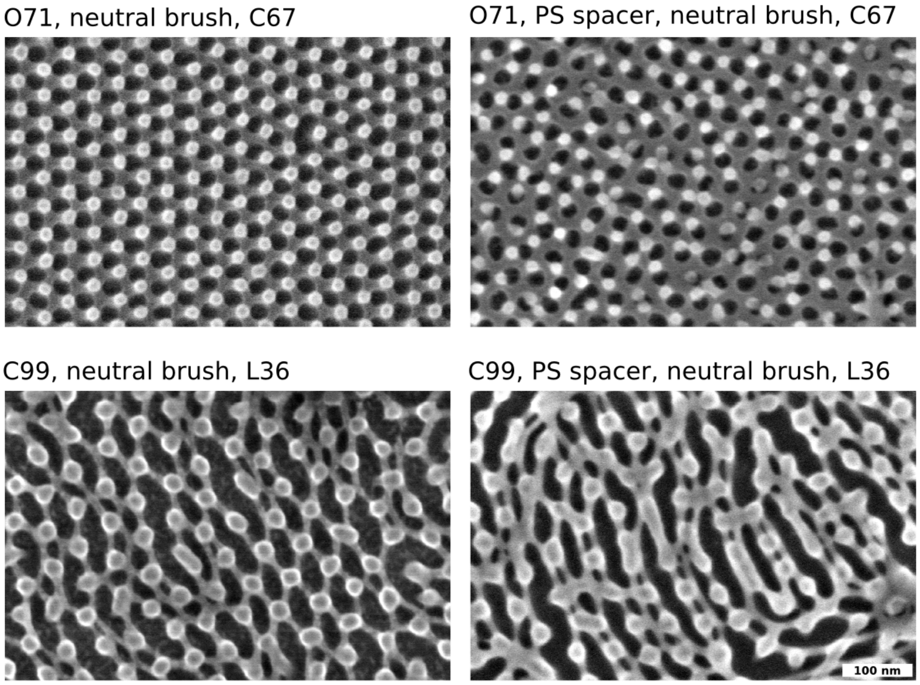

Supplementary Figure 4: Evidence of responsive templating in two-layer morphologies (images taken after final ashing of the bilayer structures). In our ordering strategy (left), the height variation of the bottom layer templates the registry of the second layer. As a control (right), a PS spacer layer (30 nm) can be applied in between the layers, which smooths-out the height variation of the first layer. In such cases, the second layer orders independently from the first. This provides evidence that it is the height variation of the first layer that controls the ordering of the second layer (enforcing a particular registry, and selecting a particular morphology). Two examples are provided: C67 registering on O71 (top), and L36 forming a criss-crossing network on C99 (bottom). In both cases, the PS spacer layer masks the templating effect.

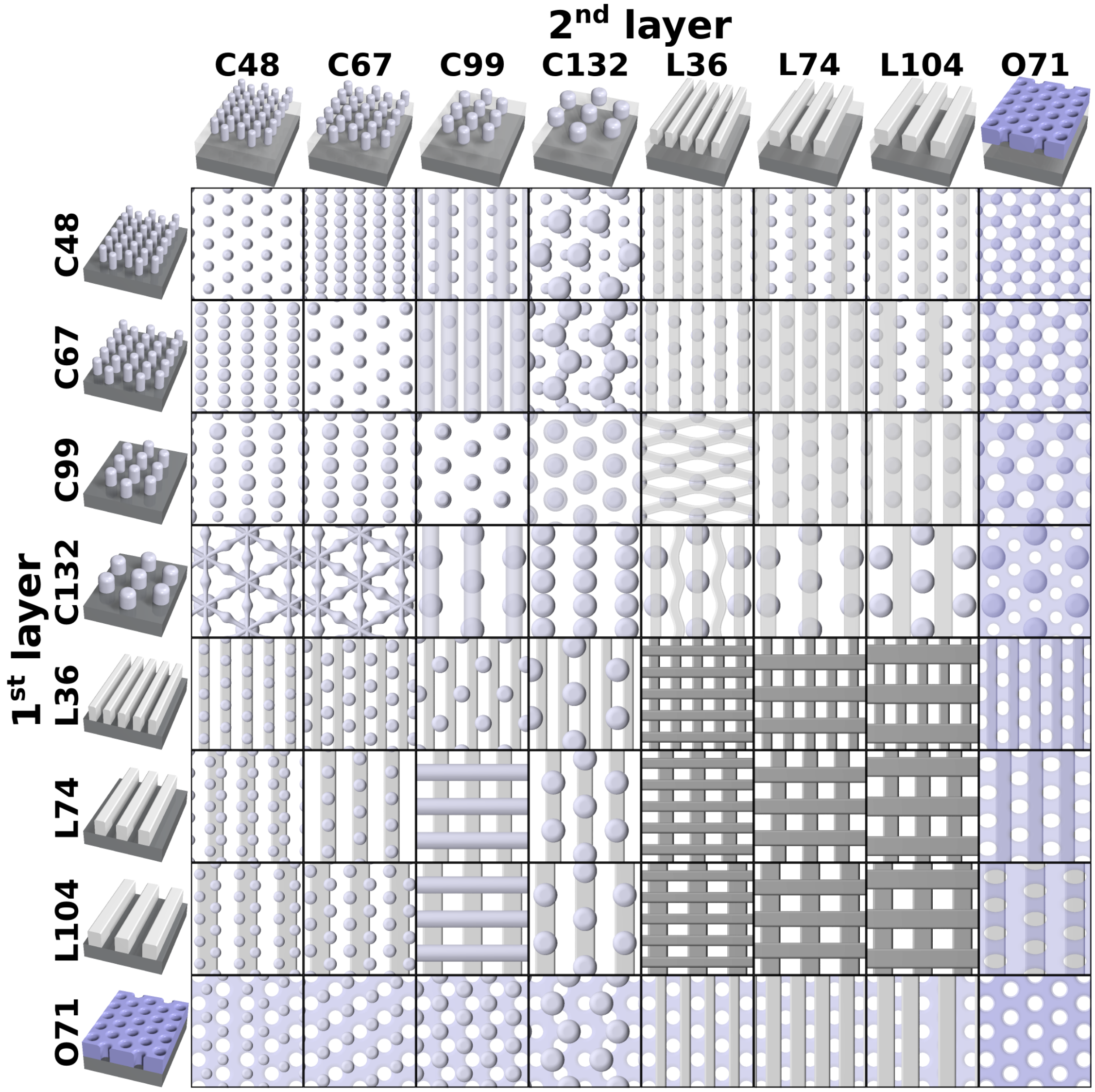

Supplementary Figure 5: Topview schematics of the idealized structures formed upon layered assembly of two block copolymer films. The structures are based upon the repeating motifs most common in the corresponding SEM data.

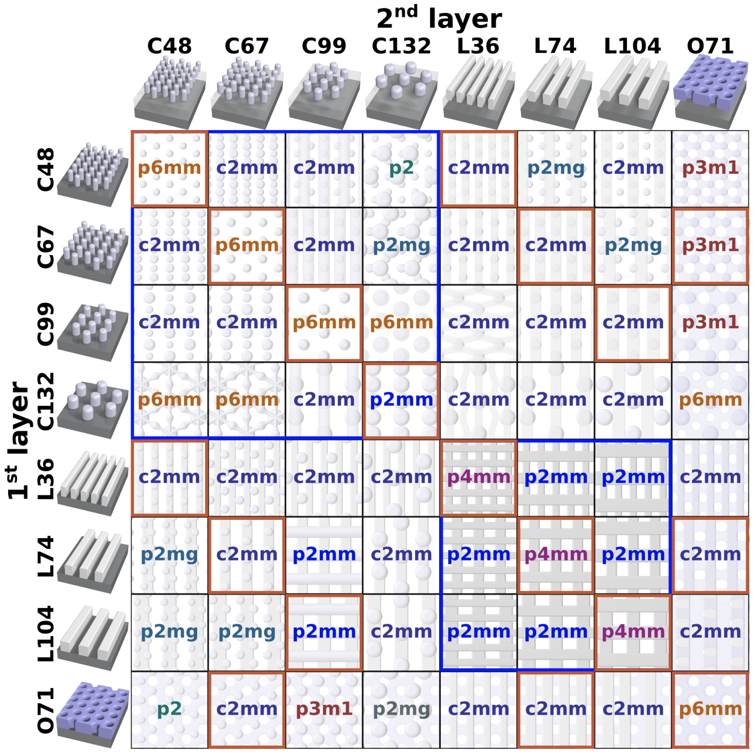

Supplementary Figure 6: Two-dimensional lattice symmetries assigned to the projection of the idealized unit cells shown in Supplementary Figure 5. The presented examples cover 7 of the 17 possible wallpaper groups. Notice that the matrix is not symmetric about the diagonal. That is, the final symmetry depends not only on the materials used, but also on the order of assembly (the self-assembly is pathway-dependent).

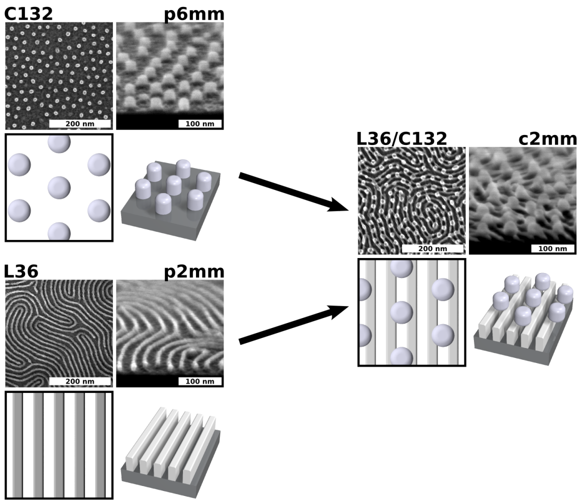

Supplementary Figure 7: In the presented example of two-layer ordering, a lamellar-forming (L36) material is used in the first layer, followed by ordering of a cylinder-forming (C132) material on top. The cylinders in the second layer position themselves in registry with the initial lamellar lines, giving rise to a symmetry (c2mm) distinct from either of the input symmetries.

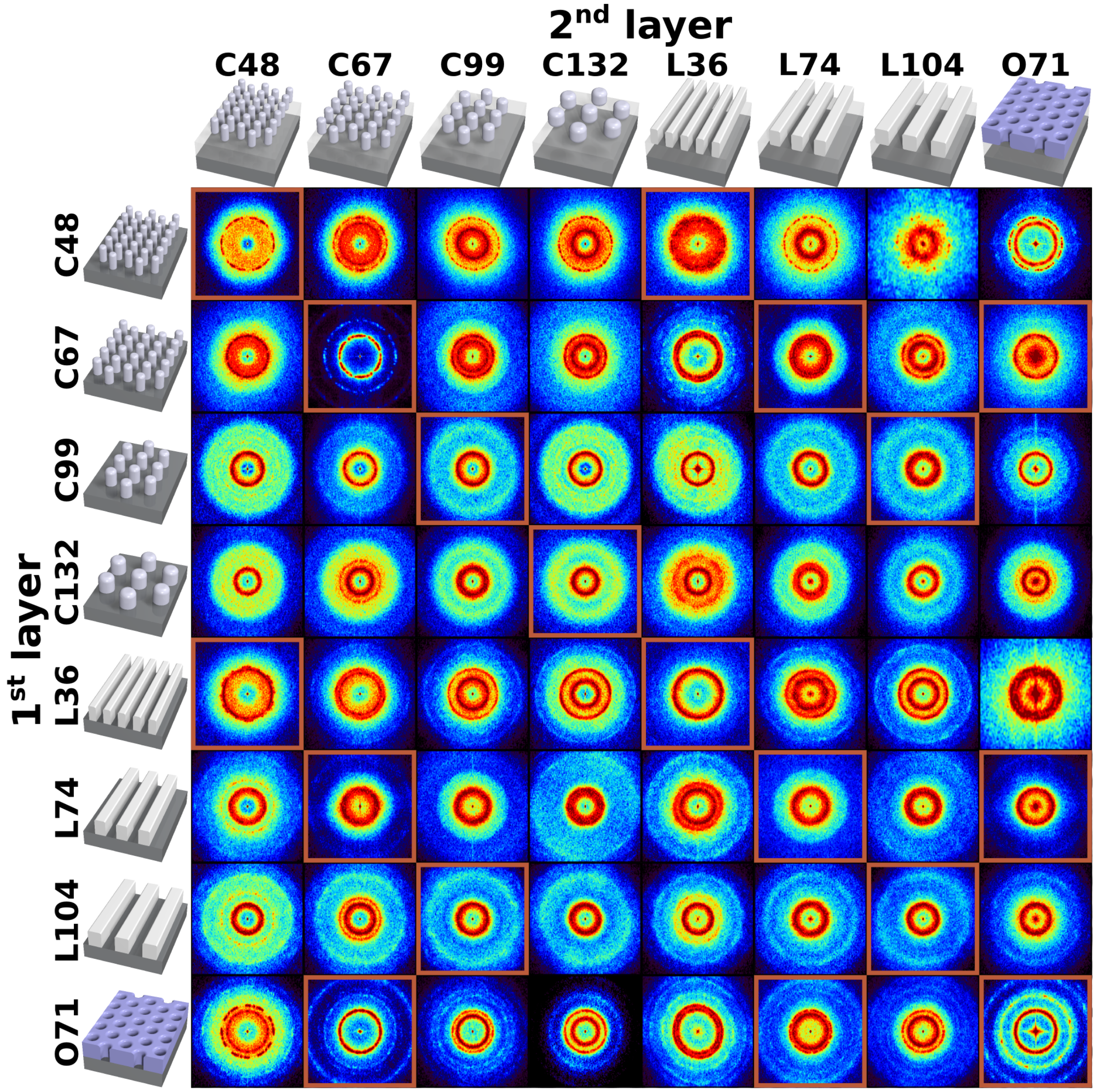

Supplementary Figure 8: Two-dimensional Fast Fourier Transforms (FFT) of wide-area SEM images ($2.540\,\mu$m $\times$ $1.905\,\mu$m). FFTs are displayed from $\pm 0.5\,\text{nm}^{-1}$ using a false-color scale.

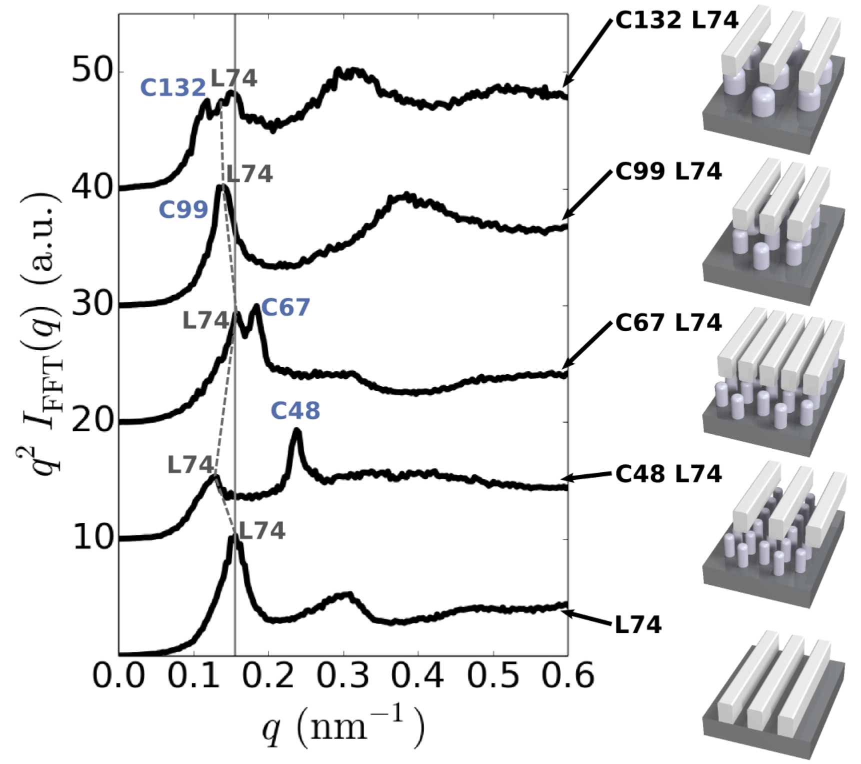

Supplementary Figure 9: One-dimensional (circular average, multiplied by q^2) curves obtained from the 2D FFT of wide-area SEM images. Curves are shifted vertically for clarity. A single layer of L74 material exhibits a peak ($q_0 \approx 0.15\,\mathrm{nm}^{-1}$) corresponding to its intrinsic repeat-spacing ($L_0 \approx 42\,\mathrm{nm}$). When this material is ordered on top of cylinder materials, it distorts its repeat-spacing to accomodate the underlying layer. E.g. L74 stretches considerably to acheive 2:1 commensurability with an underlying C48 layer.

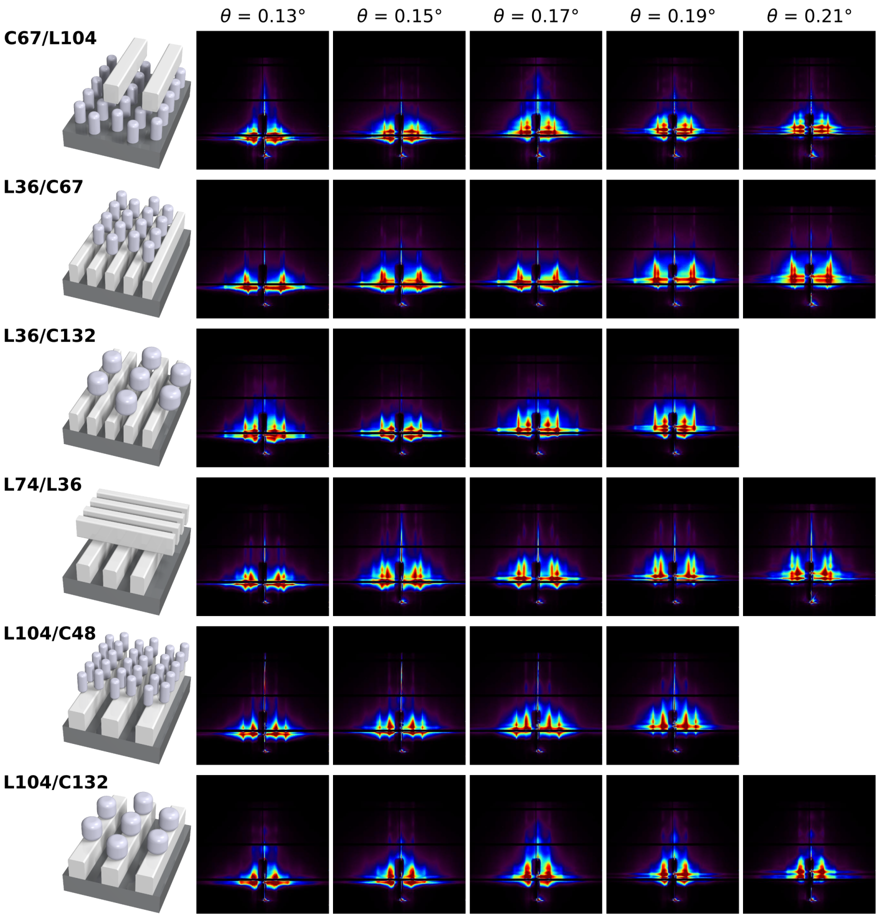

Supplementary Figure 10: Grazing-incidence small-angle x-ray scattering (GISAXS) images for a variety of two-layer nanostructures (after ashing). The columns correspond to measurements at different grazing-incidence angles (θ). Images are displayed from $-0.07\,\text{Å}^{-1}$ to $+0.07\,\text{Å}^{-1}$ in q_x, and from $-0.01\,\text{Å}^{-1}$ to $+0.14\,\text{Å}^{-1}$ in q_z. The strong scattering peaks, including higher-orders, are indicative of well-defined nanostructures over wide areas.

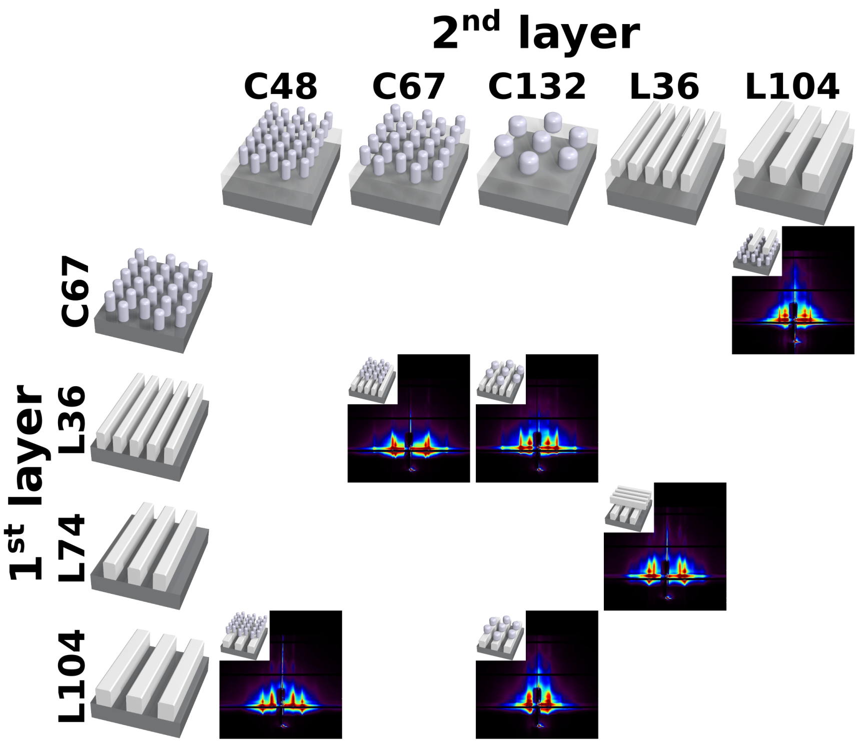

Supplementary Figure 11: Select GISAXS images of two-layer assembled nanostructures (after ashing).

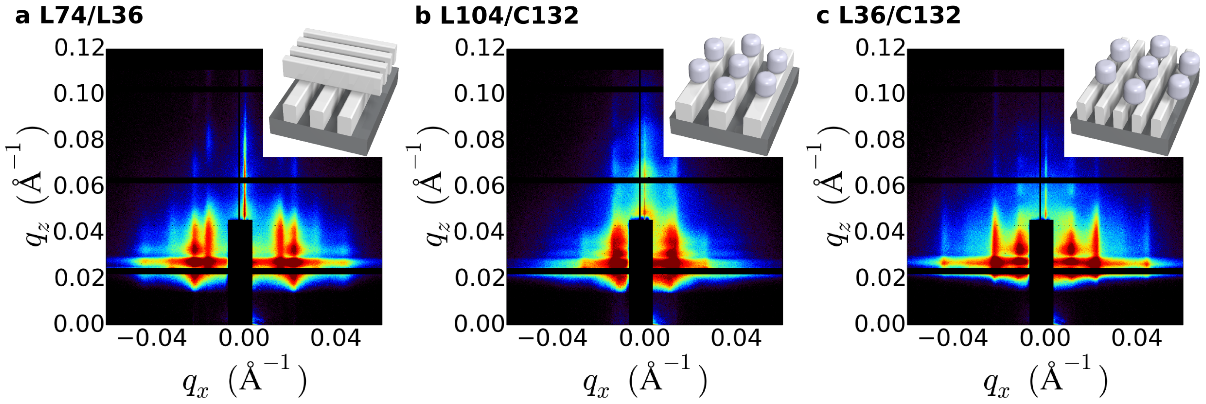

Supplementary Figure 12: (a) GISAXS pattern of two lamellar BCPs ordered on top of one another displays sets of scattering peaks that can be assigned to the individual materials. This is consistent with the SEM data, where the second-layer (L36) orients perpendicular to the first (L74) and thus orders with an unperturbed repeat-spacing (L_0). (b) GISAXS example of a system where the two materials are roughly commensurate. A single set of scattering peaks is observed, since the repeat-spacing of the top material distorts slightly to match the underlying pattern. (c) Example of two-layer ordering where the materials have very different repeat-spacings. In this case, the upper-layer (C132) material distorts its spacing to become commensurate (1:2) with the underlying material (L36). As such, the peak ascribed to the larger material (C132) appears at exactly half the q of the first-layer material. This thus implies templated ordering of the second layer.

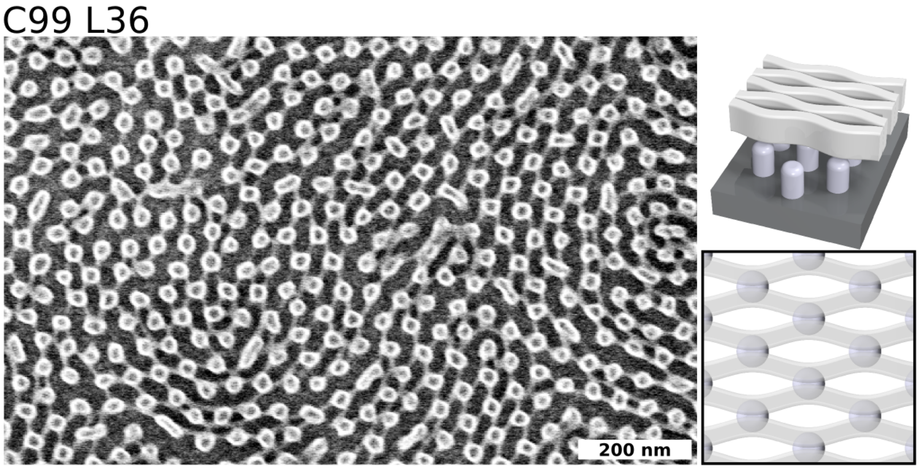

Supplementary Figure 13: SEM of a lamellar material (L36) ordering on a cylinder (C99) morphology. The upper-layer PMMA lines undulate so as to contact the underlying cylinders.

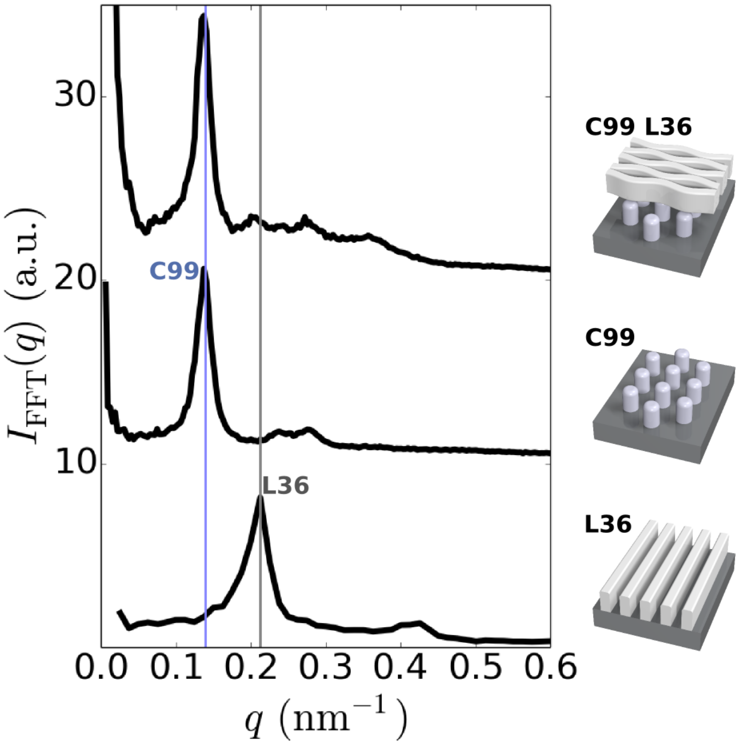

Supplementary Figure 14: One-dimensional (circular average) curves obtained from the 2D FFT of wide-area SEM images. Curves are shifted vertically for clarity. When L36 assembles on C99, it distorts to accomodate the underlying morphology. This can be seen from the complete abscence of the intrinsic L36 peak in the FFT of the two-layer system. If the second layer (L36) ordered independently from the first, the FFT of the two-layer system would instead appear as a simple summation of the FFTs of the two materials. The higher-order peaks in the two-layer system are not present in either of the input symmetries, further demonstrating the formation of a new morphology.

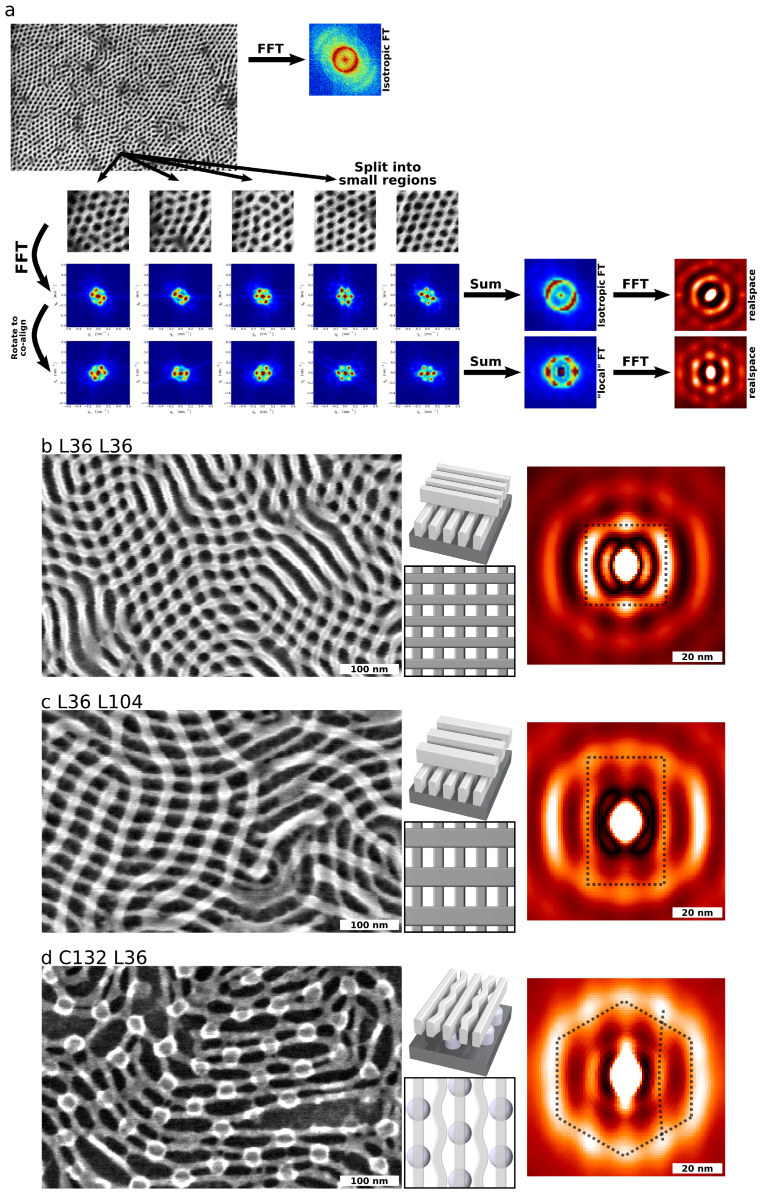

Supplementary Figure 15: (a) Image analysis is used to visualize the typical local configurations found in SEM images. For example, a cylinder-forming material generates local hexagonal order. The global FFT averages over many domains with different orientions, yielding an isotropic average. We instead compute a series of 'local' FFTs on small image sub-regions. These are mutually aligned through rotation (to bring the maximum-intensity peak along the horizontal axis). The co-aligned FFTs are summed; an inverse FFT of this yields an average of the local realspace configurations. In the example, the average hexagonal packing is recovered. (b) Local realspace for L36/L36 system displays a signature of square symmetry (dashed line is a guide to the eye). (c) The L36/L104 system instead displays rectangular symmetry. (c) The C132/L36 two-layer system exhibits local order distinct from either input materials. The average image suggests that the lamellar lines are aligned in the same direction as the hexagonal rows; moreover, there is a weak signature of the undulation of the lamellar lines.

L36 L74

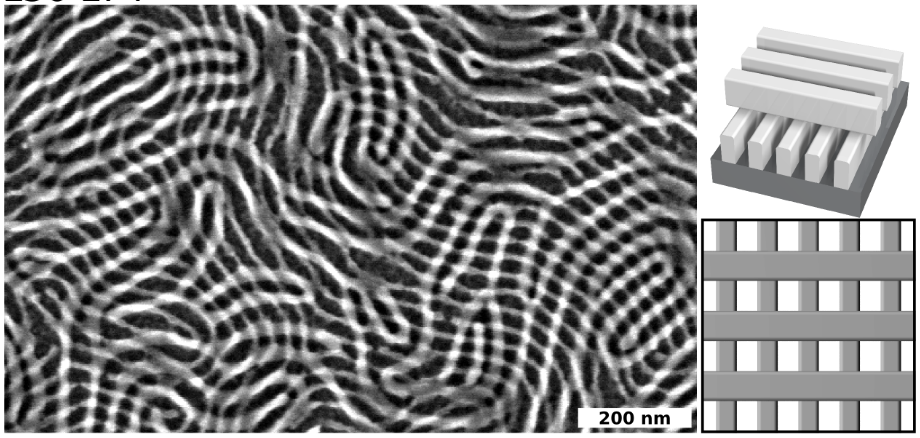

Supplementary Figure 16: SEM of a lamellar material (L74) ordering on another lamellar material (L36). The upper-layer lamellar lines generally anti-align themselves to the first layer; owing to the distinct repeat-spacings in the two layers, a rectangular symmetry is spontaneously formed.

O71 L36

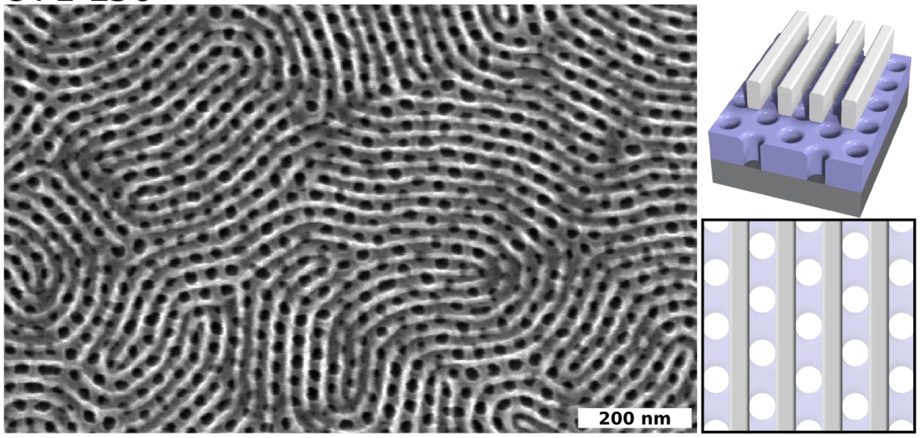

Supplementary Figure 17: SEM of a lamellar material (L36) ordering on an inverse-cylinder (O71) morphology. The upper-layer PMMA lines are positioned in-between the lower-layer rows of pores, thereby exposing these pores.

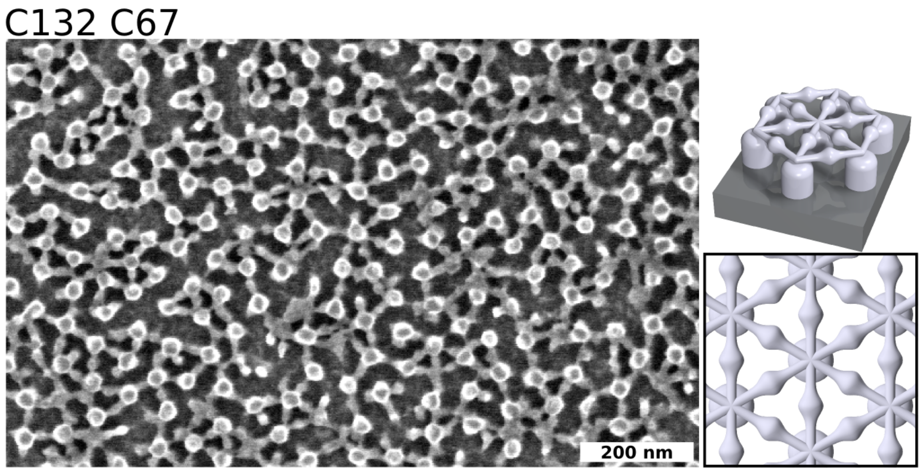

Supplementary Figure 18: SEM of a cylinder material (C67) ordering on a cylinder morphology with a larger, incommensurate, repeat-spacing (C99). The upper-layer cylinders order in a frustrated fashion, forming 'bulged bridges' between adjacent cylinders in the first layer.

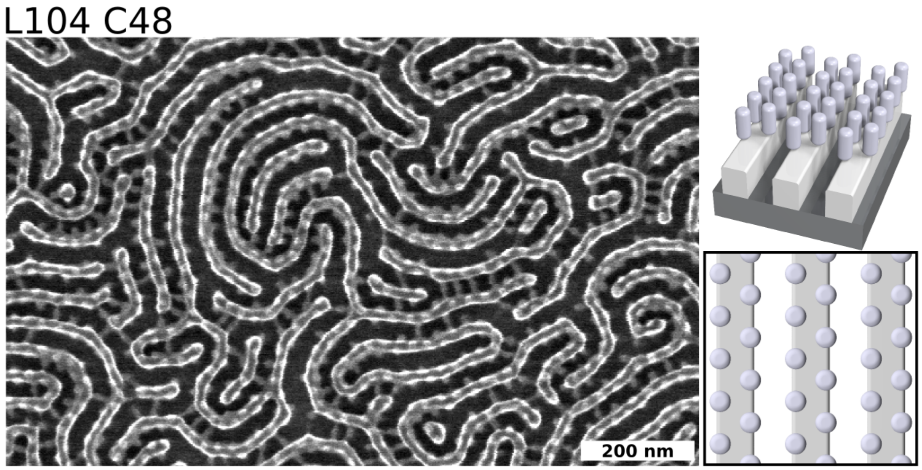

Supplementary Figure 19: SEM of a cylinder material (C48) ordering on a lamellar morphology with a larger repeat-spacing (L104). The upper-layer cylinders form a zig-zag pattern.

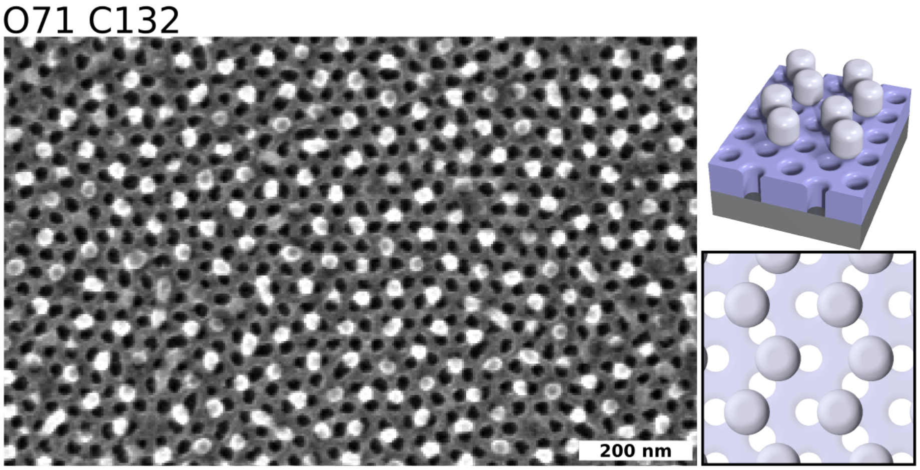

Supplementary Figure 20: SEM of a large repeat-period cylinder material (C132) ordering on an inverse-cylinder morphology (O71). The strongly incommensurate ordering induces a rotation of the second-layer hexagonal packing, demonstrating that the responsive layering phenomena controls both registry and orientation.

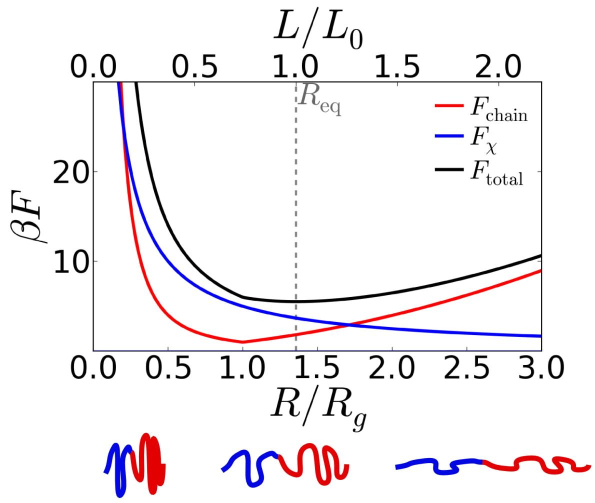

Supplementary Figure 21: Energy contributions for distortion of a polymer chain in a BCP morphology.

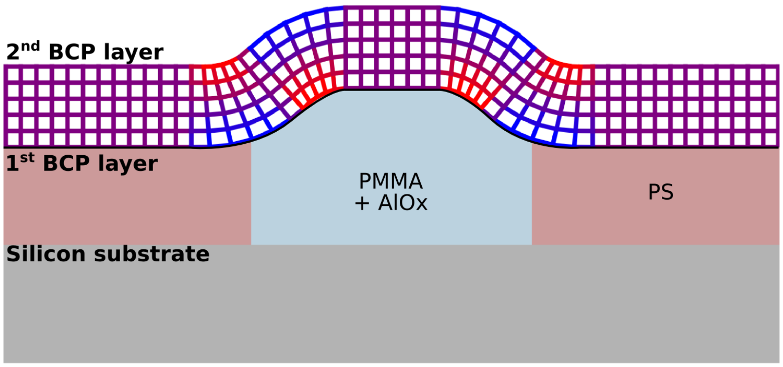

Supplementary Figure 22: A BCP film coating a topographic feature must bend to coat the substrate. This bending generates stress within the BCP morphology, with regions of compression (red) and stretching (blue; unperturbed regions shown in purple). This stress field involves a corresponding stretching or compression of the block copolymer chains.

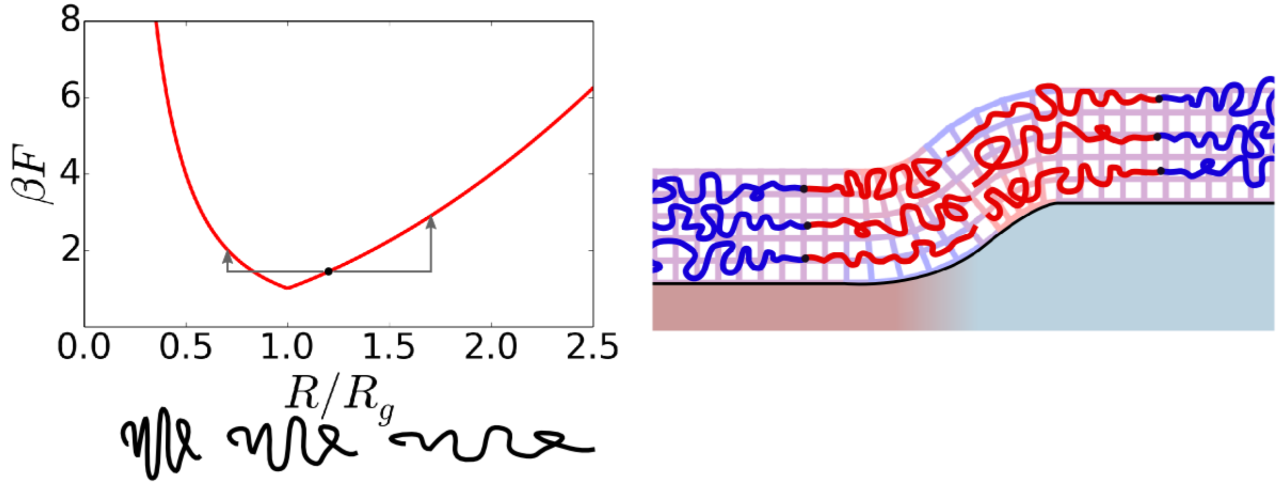

Supplementary Figure 23: The (relatively unstretched) chain segments inside a BCP domain must stretch and compress to accomodate bending. Both stretching and compressing incur an energy penalty.

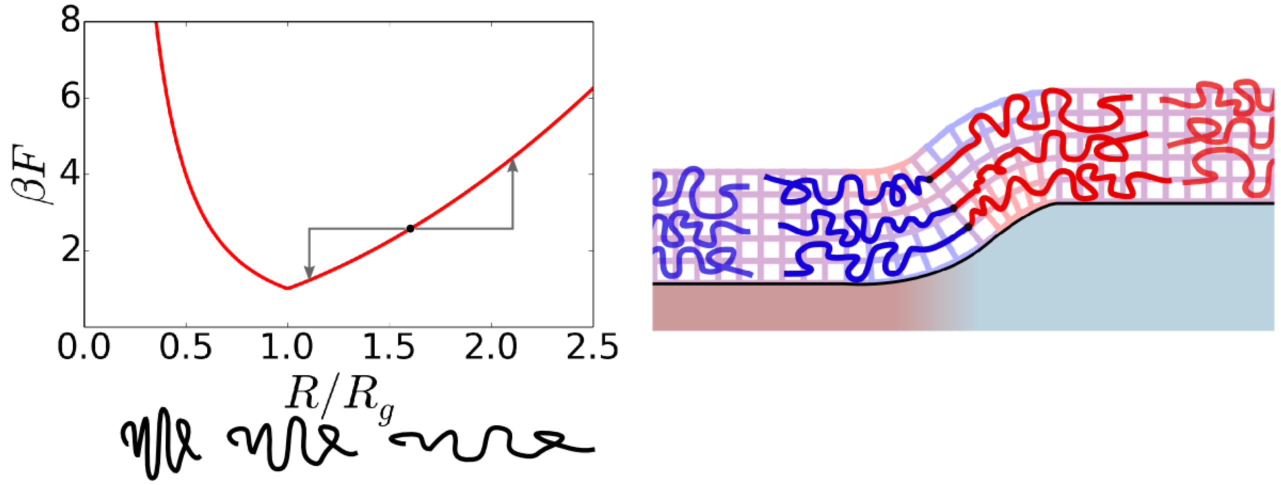

Supplementary Figure 24: The chain segments near the domain boundary in a BCP are highly stretched. When these segments accomodate film bending, some chains must stretch while others must compress (i.e. unstretch). Stretching incurs an energy penalty, whereas compressing (unstretching) involves a lowering of system energy. Overall, the energy penalty due to bending in this case is very small.

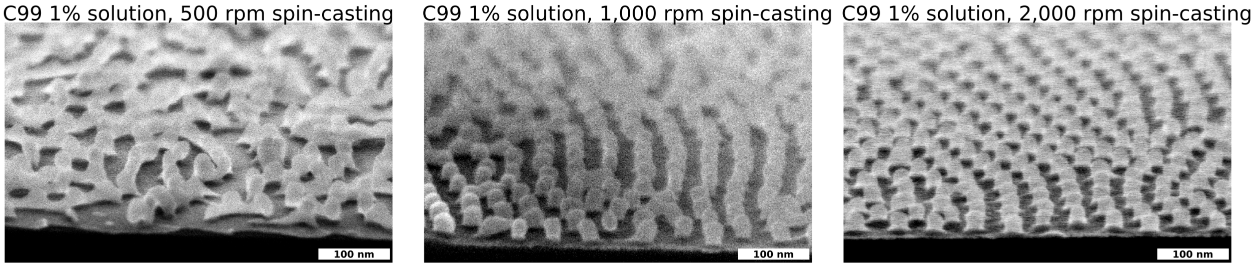

Supplementary Figure 25: SEM images demonstrating the influence of film thickness. Spin-coating rotation speed is used to tune final film thickness (higher speed generates thinner films). Film thickness influences the orientation of the morphology, as well as the ordering (e.g. defect density). Monolayer or sub-monolayer films ordering on neutral substrates tend to form well-ordered vertical orientations of the morphology (right images).

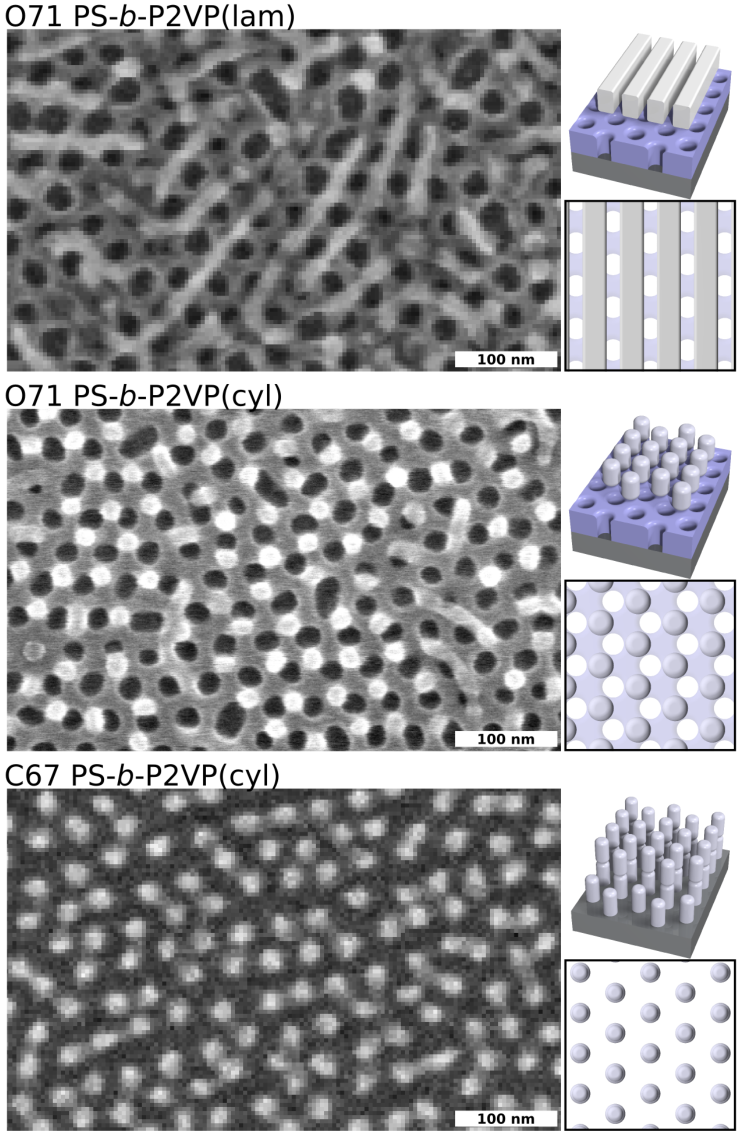

Supplementary Figure 26: SEM images demonstrating the responsive templating effect in a different BCP material: polystyrene-*block*-poly(2-vinyl pyridine) (PS-*b*-P2VP). This mixed-BCP systems exhibits similar order effects as observed for the purely PS-*b*-PMMA bilayers. The PS-*b*-P2VP material responsively registers to the underlying subtle topography.

Supplementary Discussion

We present a simple predictive model explaining the registry of BCPs ordering on weak topographic features.

Polymer Chain Distortion

The polymer chains within a BCP material can be thought of as polymer brushes ('tethered' to the block interface). The radius of gyration is:

$$R_g^2 = \frac{a^2 N}{6} \tag{1}$$

$$R_g = \frac{a N^{1/2}}{6^{1/2}} \tag{2}$$

The stretching of such a chain to a distance R incurs an energy penalty of:[1]

$$F_{\text{stretch}} = k_B T \frac{3R^2}{2a^2 N} \tag{3}$$

$$\beta F_{\text{stretch}} = \frac{1}{4}\left(\frac{R}{R_g}\right)^2 \tag{4}$$

Where we have defined $\beta = 1/k_B T$. The compression of a chain induces an energy penalty for confinement of:[1–6]

$$\beta F_{\text{confine}} = \frac{\pi^2 a^2 N}{6R^2} = \pi^2 \left(\frac{R_g}{R}\right)^2 \tag{5}$$

As expected (Supplementary Figure 21, red line), the potential exhibits roughly spring-like behavior near R_g. Combining the results (c is a constant):

$$\beta F_{\text{chain}} = \begin{cases} c\left(\frac{R}{R_g}\right)^{-2} & \text{if } R < R_g \\ c\left(\frac{R}{R_g}\right)^{2} & \text{if } R > R_g \end{cases} \tag{6}$$

Within a morphology, block copolymer do not adopt their unperturbed R_g. The driving force to minimize the interfacial area (due to the chemical-mismatch, χ) generates the morphology, which constrains possible chain configurations. Minimizing the interfacial area can be accomplished by stretching the BCP chains (thereby reducing the average area per chain). A compromise is reached, balancing chain-distortion and interfacial energies, where chains are stretched to R_{eq}. Experimentally, this chain-stretching is measured to be on the order of $R_{\text{eq}}/R_g \approx 1.2$ to 2.0.[7,8] As a rough guide, we include a $F_\chi \sim 1/R$ energetic contribution, which accounts for interfacial effects and, other driving forces in BCP ordering. The total energy per chain (F_{total}, black line) has a minimum at R_{eq}, which defines the BCP repeat-spacing (L_0). For a BCP thin film to order on top of a topographic pattern, the material must distort in some way, which will incur a corresponding distortion to the underlying polymer chains.

BCP Film Distortion

If a BCP film is ordered on a substrate with inherent topography, the BCP film will have to bend to conformally coat the substrate. Bending generates a stress field within the BCP material, which

cannot be entirely relaxed (without disrupting the morphology; e.g. increasing the inter-block interfacial area). Bending involves a combination of stretching and compression (Supplementary Figure 22). The regions of compressed or stretched material will involve a corresponding compression or stretching of the polymer chains forming the morphology.

BCP Chains

The chains within a BCP mesophase are known to be stretched. However, this stretching is not uniform along the chain length. In particular, the segments near the inter-block interface are preferentially stretched, as compared to the segments closer to the center of the chain length (which are closer to the unperturbed chain conformation).[9] As a result, the aforementioned bending (compression and stretching) influences the domain boundary part of the BCP morphology differently than the 'bulk' region (regions farther from the domain boundary). Bending of the 'bulk' region involves stretching some BCP chains, and compressing other chains. Both of these incur energy penalties (Supplementary Figure 23). On the other hand, bending of the domain boundary involves distortion of chains which are already highly stretched. Stretching these chains further involves an energy penalty. However, compressing these chains is better described as 'unstretching' and thus involves a net lowering of system energy (Supplementary Figure 24). Overall, the energy penalty for distorting the BCP domain boundary is thus considerably lower than distortion other regions of the morphology. This results in the BCP organizing so as to maximize the overlap between substrate height modulations, and the domain boundary region of the morphology (since this overall lowers the chain distortion energy penalty).

Supplementary References

[1] Skvortsov, A. M.; Klushin, L. I.; Birshtein, T. M. Stretching and compression of a macromolecule under different modes of mechanical manupulations *Polymer Science Series A* **51**, 469–491 (2009).

[2] Khokhlov, A. R.; Semenov, A. N. Liquid-crystalline ordering in the solution of long persistent chains *Physica A: Statistical Mechanics and its Applications* **108**, 546–556 (1981).

[3] Khokhlov, A. R.; Semenov, A. N. Liquid-crystalline ordering in the solution of partially flexible macromolecules *Physica A: Statistical Mechanics and its Applications* **112**, 605–614 (1982).

[4] Hsu, H.-P.; Grassberger, P. Polymers confined between two parallel plane walls *The Journal of Chemical Physics* **120**, 2034–2041 (2004).

[5] Hsu, H.-P.; Binder, K. Semi-flexible polymer chains in quasi-one-dimensional confinement: a Monte Carlo study on the square lattice *Soft Matter* **9**, 10512–10521 (2013).

[6] Smyda, M. R.; Harvey, S. C. The Entropic Cost of Polymer Confinement *The Journal of Physical Chemistry B* **116**, 10928–10934 (2012).

[7] Almdal, K.; Rosedale, J. H.; Bates, F. S.; Wignall, G. D.; Fredrickson, G. H. Gaussian- to stretched-coil transition in block copolymer melts *Physical Review Letters* **65**, 1112–1115 (1990).

[8] Antonietti, M.; Heinz, S.; Schmidt, M.; Rosenauer, C. Determination of the Micelle Architecture of Polystyrene/Poly(4-vinylpyridine) Block Copolymers in Dilute Solution *Macromolecules* **27**, 3276–3281 (1994).

[9] Forrey, C.; Yager, K. G.; Broadaway, S. P. Molecular Dynamics Study of the Role of the Free Surface on Block Copolymer Thin Film Morphology and Alignment *ACS Nano* **5**, 2895–2907 (2011).
